# Therapeutic alliance with psychotherapist versus dietician: a pilot study of eating disorder treatment in a multidisciplinary team during the COVID-19 pandemic

**DOI:** 10.3389/fpsyt.2023.1267676

**Published:** 2024-01-29

**Authors:** Roni Elran-Barak, Rinat Grundman-Shem Tov, Eynat Zubery, Yael D. Lewis

**Affiliations:** ^1^School of Public Health, University of Haifa, Haifa, Israel; ^2^Hadarim Eating Disorders Treatment Center, Shalvata Mental Health Center, Hod Hasharon, Israel; ^3^Faculty of Medicine, Tel Aviv University, Tel Aviv, Israel

**Keywords:** eating disorders, therapeutic alliance, multidisciplinary treatment, dietician, COVID-19

## Abstract

Little is known about the therapeutic alliance (TA) formed with different professionals in multidisciplinary eating disorder (ED) treatment, particularly in the context of online treatment during the COVID-19 pandemic. We aimed to conduct a pilot study during the COVID-19 pandemic examining characteristics of patients’ TA with their dieticians and psychotherapists, associations between patients’ and therapists’ views of the TA, and relationships between psychological characteristics and TAs. Sixty-three patients with EDs and their treating psychotherapists and dieticians were surveyed during the COVID-19 pandemic using the Working Alliance Inventory (WAI-S). Spearman correlation tests were used to examine associations between variables. Positive associations were found between the TAs examined. Concordance was stronger in patient–dietician dyads than in patient–psychotherapist dyads. Severe ED psychopathology was associated with weaker TA (bond subscale). General psychopathology was associated with weaker TA with the dietician (task subscale). Given that several differences were found between the TAs of treatment dyads, further longitudinal studies are needed to validate our pilot findings and to investigate multidisciplinary TAs and their impact on treatment outcomes in online ED treatment settings during the COVID-19 pandemic, as well as in other treatment settings (e.g., in-person settings). This study will contribute to a deeper understanding of the dynamics of TAs in multidisciplinary ED treatment and inform the development of more effective interventions.

## Introduction

Eating disorders (EDs) are complex psychiatric disorders (1) that involve a range of maladaptive eating behaviors, such as restrictive eating, excessive exercising, binge eating, and purging. Additionally, individuals with EDs often have distorted attitudes toward food, weight, and body image, such as an intense fear of gaining weight, a preoccupation with body shape and size, and a persistent dissatisfaction with their appearance, regardless of their actual weight or physical appearance (2, 3). These behaviors and attitudes can significantly impact an individual’s physical health, emotional wellbeing, and overall quality of life, emphasizing the need for comprehensive and specialized treatment approaches delivered by a multidisciplinary team to address intricate medical, nutritional, familial, and psychological aspects involved (4).

The study of the therapeutic alliance (TA) in EDs has to date been limited and inconsistent in scope and methods (5, 6). However, researchers have confirmed its centrality in the therapeutic process and link to treatment outcomes, similar to that which occurs in the context of other disorders (7). A meta-analysis (6) of the relationship between TA and treatment outcome in EDs implies that there is a significant association between early symptom improvement and subsequent alliance quality, as well as a relationship between alliance ratings and subsequent symptom reduction in ED treatments. Nevertheless, to the best of our knowledge, the TA of patients with different therapists in a multidisciplinary ED team has only been examined in one previous study (8); in that study, incongruencies between the professions as well as gaps between patients’ and therapists’ perceptions were revealed.

Eating disorder treatment requires health professionals from various disciplines to work together as a team (9, 10). However, forming TAs within a multidisciplinary team can be challenging due to the patient’s need to establish and maintain multiple TAs with different members of the multidisciplinary team (11). Each member of the team brings unique expertise and perspectives, and patients can feel that they are more comfortable with one team member than with the other (12). Therefore, understanding the dynamics and quality of these different alliances can contribute to improving the effectiveness and coordination of care in multidisciplinary ED treatment settings.

The current study focuses on the TA formed between patients and two key members of the ED treatment team: the psychotherapist and the dietician. While the role of the psychotherapist in the team is primarily focused on addressing the psychological and emotional aspects of the ED (13, 14), the dietician is responsible for managing the nutritional and behavioral (food-related) aspects of treatment, creating individualized meal plans, and educating patients about balanced and healthy eating habits (15, 16). Although a nutritional component is recognized as crucial in ED treatments (17), little evidence exists to guide the ED dietetic intervention, and the inclusion of dieticians in different treatment models is not always implemented (15, 16). In the treatment model described in the current study, there is an inclusion of weekly individual dietetic symptom-focused treatment prior to and in parallel with weekly individual psychotherapy, while medical, psychiatric, and family therapy interventions accompany psychotherapy and nutritional therapy according to patients’ needs. Therefore, we sought to study the TA that patients formed with their psychotherapist and dietician with the goal of improving understanding of the nature and dynamics of the TA within an ED multidisciplinary team.

Exposure to stressors related to COVID-19 has been shown to result in heightened psychological distress, depression, and anxiety symptoms (18). This marked impact of COVID-19 on mental health, coupled with ED risk factor stressors, including disrupted food routines, movement and exercise constraints, and decreased support imposed an increased risk for the development and deterioration of EDs (19, 20) which was later confirmed in the growing rates of ED cases seeking treatment during the pandemic (21). A recent systematic review included 53 COVID-19 studies in EDs and found an increase in ED admissions and an increase in ED symptoms, anxiety, depression, and changes to BMI in ED patients during the pandemic (22).

The imposed lockdown and self-isolation measures in the early COVID-19 outbreak have necessitated a rapid shift to online treatment, potentially posing challenges for alliance formation and maintenance (23, 24). In this context, we aimed to investigate the TA between patients and their dieticians and psychotherapists, as perceived by both parties, during the first COVID-19 lockdown. Understanding TAs during the COVID-19 global pandemic is crucial (21) as the abrupt transition to online treatment may have presented additional obstacles in forming and maintaining alliances (23, 24).

Specifically, we aimed to address the following four objectives in the context of online treatment during the COVID-19 pandemic:

(1) Strength of the TAs:

a. To assess the strength of the TAs formed between patients and their dieticians, as viewed by both sides.
b. To assess the strength of the TAs formed between patients and their psychotherapists, as viewed by both sides.

(2) Agreement or discrepancy in the TA:

a. To examine the levels of agreement between patients and psychotherapists regarding their perspectives of the TAs, as measured via the associations between the TAs, as viewed by both sides.
b. To examine the levels of agreement between patients and dieticians regarding their perspectives of the TAs, as measured via the associations between the TAs, as viewed by both sides.

(3) Associations between the TAs:

a. To examine associations between the TAs formed with dieticians and psychotherapists.

(4) Psychological characteristics and the TA:

a. To examine associations between patients’ psychological characteristics and their TAs with their dietician and psychotherapist.

## Methods

Addressing the four objectives detailed above could provide valuable insights into the nature of therapeutic alliances within multidisciplinary ED treatments in an online setting. It may further help to identify the specific role that each team member may play and facilitate the tailoring of interventions to meet the individual needs of each patient.

### Procedure

The Institutional Review Board (IRB) at Shalvata Mental Health Center approved the study in a special meeting convened to discuss COVID-19-related studies. Data were collected between mid-April and mid-May 2020, a time in which, due to the COVID-19 social distancing restrictions, all non-urgent services in the clinic (including psychotherapy and dietetic and psychiatric consultations) were switched from in-person to online platforms. Approximately 95% of patients in the treatment center were invited to participate in the study, and approximately 80% of them consented. Patients who consented to participate received a personal link to an anonymous survey via the Qualtrics online platform. The dietician and psychotherapist of each participant also received a personal link to assess their TA with this patient. Dieticians and psychotherapists did not have access to the patient’s survey as well as to the survey of each other. This ensured that their assessments of the TA were independent and based solely on their own observations and interactions with the patient. Eating disorder diagnoses were decided in accordance with the DSM-5 and in clinic discussions held among a multidisciplinary team (psychiatrist, clinical social worker, and dietician). The last measured BMI was collected from the patient files.

### Participants

Participants included 63 patients recruited from the patient population of Hadarim Eating Disorders Treatment Center in Kfar Saba, a part of the Shavata Mental Health Center. Table 1 shows the demographic and clinical characteristics of participants. The age of the participants ranged from 12 to 56 years, with a mean age of 27.25 years (SD = 11.47). Among the participants, 57 (90.50%) identified as women, while 6 (9.50%) identified as men. In terms of marital status, 38 (60.32%) participants reported being single, 23 (36.51%) were married or living with a partner, and 2 (3.17%) were divorced. Regarding education level, 17 (27.0%) participants were students at the time of study, 15 (23.80%) had completed high school, 8 (12.70%) were university students, and 23 (36.50%) were university/college graduates.

**Table 1:** Demographic and clinical characteristics (*N* = 63).

Demographic characteristics	
Age, years: M (SD), R	27.25 (11.47), [12–56]
Gender: n (%)	
Women	57 (90.50)
Men	6 (9.50)
Marital status: n (%)	
Single	38 (60.32)
Married/living with partner	23 (36.51)
Divorced	2 (3.17)
Other	
Level of education: n (%)	
Student	17 (27.0)
High school graduate	15 (23.80)
University student	8 (12.70)
University/college graduate	23 (36.50)
Clinical characteristics	
Diagnosis: n (%)	
Anorexia nervosa	24 (38.10)
Bulimia nervosa	20 (31.75)
Binge eating disorder	16 (25.40)
Other ED	3 (4.75)
Age of ED onset: M (SD), R	15.39 (4.99), [12–56]
BMI (last measured), kg/m^2^: M (SD), R	24.82 (7.40), [16.36–52.61]
Duration of treatment, days: M (SD), R	317 (195), [42–909]
Past ED hospitalization: n (%)	14 (22.22)
ED psychopathology (EDE-Q)^1^: M (SD), R	3.51 (1.35), [0.58–6.06]
Psychiatric comorbidity (DASS)^2^: M (SD), R	13.83 (9.17), [0.00–38.67]

M, mean; SD, standard deviation; R, range; BMI, body mass index; ED, eating disorder.

1 Eating disorder examination questionnaire.

2 Depression, anxiety, and stress scale.

In terms of clinical characteristics, the participants had various diagnoses, with 24 (38.10%) being diagnosed with anorexia nervosa, 20 (31.75%) with bulimia nervosa, 16 (25.40%) with binge eating disorder, and 3 (4.75%) with other forms of EDs. The average age of onset for their ED was 15.39 years (SD = 4.99), ranging from 12 to 56 years. The participants’ body mass index (BMI) at the last measured point ranged from 16.36 to 52.61 kg/m^2^, with a mean of 24.82 kg/m^2^ (SD = 7.40). The duration of treatment for the participants ranged from 42 to 909 days, with a mean of 317 days (SD = 195). Additionally, 14 (22.22%) participants reported having been hospitalized for their ED in the past.

### Statistical analyses

All statistical analyses were conducted using SPSS-25. Statistical significance was set at a value of *p* of <0.05. To examine the associations between TAs and between TAs and participants’ clinical characteristics, Spearman correlation coefficients were calculated using R software.

### Measures

**Working alliance** was assessed using the short version of the **Working Alliance Inventory (WAI)**, a widely used instrument that includes three subscales examining the agreement between patient and therapist on the **goal** of therapy and on the **task** of therapy, as well as the development of the therapist–patient **bond** (25). The Hebrew version of the WAI has also been used extensively (26) with good internal consistency (α range: 0.73–0.84).

For each patient, the questionnaire was completed four times as follows:

by the patient toward their dietician (*α* = 0.791)
by the patient toward their psychotherapist (*α* = 0.817)
by the dieticians toward the patient (*α* = 0.841)
by the psychotherapists toward the patient (total: *α* = 0.732)

**Clinical characteristics**, including ED psychopathology and comorbidities, were measured using the Hebrew versions of two questionnaires. **The Eating Disorder Examination Questionnaire (EDE-Q)** was used to assess ED symptoms (27) and the **Depression Anxiety and Stress Scales (DASS)**—version 21 was used to examine general psychopathology (28). The EDE-Q has been widely used in the study of EDs (29), and the Hebrew version has demonstrated good convergent validity (30). The internal consistency of the EDE-Q in the present study was *α* = 0.93. The mean score of the EDE-Q score was 3.51 (SD = 1.30), in line with previously described clinical ED samples (31, 32) and above 95% of the general population (33). The DASS has demonstrated high reliability (34). In our study, internal consistency was *α* = 0.95, comparable with previously reported scores in the Hebrew version (35).

## Results

Table 2 addresses the first aim of the study. The therapeutic alliance between patients and their dietician and psychotherapist was measured using three dimensions: goal, bond, and task which are represented in three separate subscales. Table 2 shows that the TAs as reported by both patients and staff were relatively strong (min = 4.58 + 1.33, max = 5.62 + 0.87). Patient–dietician ratings (i.e., patient’s perception of the TA with the dietician) indicated a mean score of 5.19 (SD = 1.34) for goal, 5.59 (SD = 1.24) for bond, and 5.16 (SD = 1.51) for task. Patient–psychotherapist ratings (i.e., patient’s perception of the TA with the psychotherapist) showed a mean score of 5.37 (SD = 1.11) for goal, 5.42 (SD = 1.25) for bond, and 5.38 (SD = 1.28) for task. The dietician–patient ratings (dietician’s perception of the TA with the patient) yielded a mean score of 5.36 (SD = 1.64) for goal, 5.52 (SD = 1.51) for bond, and 5.27 (SD = 1.69) for task. The psychotherapist–patient ratings (psychotherapist’s perception of the TA with the patient) yielded a mean score of 4.95 (SD = 1.27) for goal, 5.62 (SD = 0.87) for bond, and 4.58 (SD = 1.33) for task.

**Table 2:** Therapeutic alliances among patients with eating disorders (*N* = 63).

Therapeutic alliance	Goal	Bond	Task
Patient to dietician^1^: M (SD), R	5.19 (1.34), [2.75, 7.00]	5.59 (1.24), [1.75, 7.00]	5.16 (1.51), [1.25, 7.00]
Patient to psychotherapist^2^: M (SD), R	5.37 (1.11), [3.25, 7.00]	5.42 (1.25), [2.00, 7.00]	5.38 (1.28), [1.75, 7.00]
Dietician to patient^3^: M (SD), R	5.36 (1.64), [2.75, 7.00]	5.52 (1.51), [3.50, 7.00]	5.27 (1.69), [1.00, 7.00]
Psychotherapist to patient^4^: M (SD), R	4.95 (1.27), [1.00, 7.00]	5.62 (0.87), [1.00, 7.00]	4.58 (1.33), [1.00, 7.00]

M, mean; SD, standard deviation; R, range.

1 Patient to dietician: patient’s perception of the therapeutic alliance with the dietician.

2 Patient to psychotherapist: patient’s perception of the therapeutic alliance with the psychotherapist.

3 Dietician to patient: dietician’s perception of the therapeutic alliance with the patient.

4 Psychotherapist to patient: psychotherapist’s perception of the therapeutic alliance with the patient.

**Figure 1:**
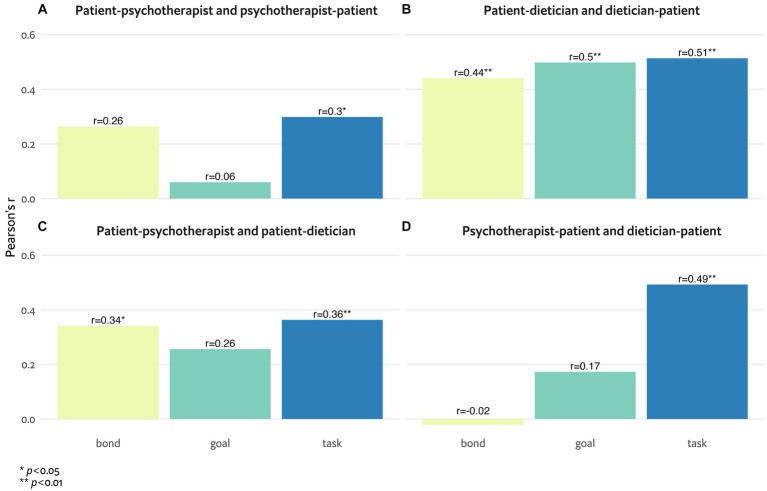
**(A-D)** Associations between therapeutic alliance perceptions of patients, psychotherapists and dieticians, measured by the bond, goal and task subscales of the working alliance inventory.

Figures 1A–D demonstrate associations between the TA as viewed by both sides. While Figures 1A,B address the second aim of the study, Figures 1C,D address the third aim of the study. Figure 1A presents the associations between patient–psychotherapist (i.e., patient’s perception of the alliance with the psychotherapist) and psychotherapist–patient (i.e., psychotherapist’s perception of the alliance with the patient) TAs and suggests significant associations in the task subscale (*r* = 0.32, *p* < 0.05). Figure 1B presents the associations between patient–dietician (i.e., patient’s perception of the alliance with the dietician) and dietician–patient (i.e., dietician’s perception of the alliance with the patient) TAs and suggests significant associations in all subscales. Figure 1C presents the associations between patient–psychotherapist (i.e., patient’s perception of the alliance with the psychotherapist) and patient–dietician (i.e., patient’s perception of the alliance with the dietician) TAs and suggests significant associations in all subscales. Figure 1D presents the associations between psychotherapist–patient (i.e., psychotherapist’s perception of the alliance with the patient) and dietician–patient (i.e., dietician’s perception of the alliance with the patient) TAs and suggests significant associations in the task subscale (*r* = 0.35, *p* < 0.05).

Psychological characteristics and their associations with TA are shown in detail in Table 3 (the fourth aim of the study). Participants with more severe ED psychopathology reported a weaker TA with their psychotherapist and their dietician (bond only). Participants with more psychiatric comorbidity had a weaker TA with their dietician (task only).

**Table 3:** Associations between patients’ characteristics and therapeutic alliance with their dietician and psychotherapist.

		Eating disorder psychopathology (EDE-Q)^1^	Psychiatric comorbidity (DASS)^2^
Patient–psychotherapist^3^	Goal	***r* = −0.303, *p* = 0.023**	*r* = −0.137, *p* = 0.315
	Bond	***r* = −0.294, *p* = 0.028**	*r* = −0.150, *p* = 0.270
	Task	***r* = −0.293, *p* = 0.029**	*r* = −0.224, *p* = 0.097
Patient–dietician^3^	Goal	*r* = −0.129, *p* = 0.329	*r* = −0.180, *p* = 0.173
	Bond	***r* = −0.307, *p* = 0.018**	*r* = −0.253, *p* = 0.053
	Task	*r* = −0.224, *p* = 0.088	***r* = −0.318, *p* = 0.014**

Significant values (*p* < 0.05) appear in bold.

1 Eating disorder examination questionnaire.

2 Depression, anxiety, and stress scale.

3 Working alliance inventory—short version.

## Discussion

This pilot study, conducted during the COVID-19 pandemic and in an online treatment setting, aimed to explore the nature of the TAs that patients with EDs form with their psychotherapists and dieticians in multidisciplinary treatment. This study represents one of the first attempts to examine the concordance between patient and therapist views of the alliance and also incorporate the perspectives of both dieticians and psychotherapists. Overall, all TAs were assessed to be relatively strong, which aligns with the range of findings in previous ED studies (36, 37). Specifically, agreement between the patient’s and the therapist’s views of the TA was stronger in patient–dietician dyads compared to patient–psychotherapist dyads. Notably, severe ED psychopathology was associated with a weaker bond subscale alliance with both the psychotherapist and the dietician, while general psychopathology was associated with a weaker task subscale alliance with the dietician.

Significant correlations were found between patient and psychotherapist for the task component of the TA but not goal or bond (Figure 1A), whereas correlations between patients and dieticians were more robust and significant across all three TA components (Figure 1B). These stronger correlations between the patient–dietician TA and the dietician–patient TA, relative to the patient–psychotherapist TA and the psychotherapist–patient TA, suggest a stronger agreement between how the dietician and the patient perceived their bond, relative to the psychotherapist and the patient. Stronger alliance congruence between patient and therapist has been shown in prior studies to be related to better treatment outcomes and symptom relief (38–40), a finding that highlights its importance. The only study, to our knowledge, in which TA perceptions of patients and therapists in an ED multidisciplinary team were examined revealed few correlations between therapists and patients compared with those in our study, possibly reflecting the smaller sample (*n* = 21 vs. *n* = 63 in our study) and a more severe and resistant ED inpatient population (8).

When treating EDs in a multidisciplinary team, the nutritional counseling focuses on symptom reduction (e.g., meal plan and identifying triggers for binge eating), whereas the psychotherapy centers on the emotional underlying issues. This is, to the best of our knowledge, the first study examining alliances that the same patient forms with two therapists under two complementary treatment strategies. Research regarding the influence of the type of therapy conducted (i.e., the treatment strategy) on the TA has produced inconsistent results. A meta-analysis in which patient–therapist perspectives of the working alliance across several aspects were examined revealed weaker associations between alliance perceptions in CBT vs. psychodynamic therapy, although this analysis was based on small samples (41). In contrast, Raue et al. (42) assessed alliance with independent raters observing therapy sessions and found stronger TAs in CBT compared with psychodynamic therapy. These results are similar to ours, perhaps as for ED patients a clearly defined focus (i.e., symptom reduction) may facilitate the forming of a TA with the dietician compared with the psychotherapist who focuses on underlying issues.

Significant correlations were found between the patient’s perception of the alliance with the psychotherapist and the dietician in all components (Figure 1C), whereas the only significant correlation that emerged from the psychotherapist’s and dietician’s perspectives was for the task component (Figure 1D), suggesting disagreement between how the psychotherapists and dieticians perceived the patients. Comparable with our results, Gallop et al. (8) described no congruence among the differing disciplines’ alliance perceptions. Clinical guidelines repeatedly emphasize the need for treatment to be conducted in a multidisciplinary setting (43). Our findings highlight the complexity of this treatment setting, particularly in an ambulatory setting where disconnects and conflicts often arise (44). Regardless of which evidence-based treatment strategy is attempted, different treatment perspectives should be openly deliberated to promote team collaboration (8)—a crucial factor when dieticians and mental health professionals are treating ED patients and families under severe stress (17). Moreover, reducing patient–staff incongruencies and promoting agreement in the TA are beneficial to treatment outcomes (6). One possible way to better guide the therapeutic process beyond staff communication can be incorporating routine alliance monitoring (40).

Regarding factors associated with TA scores, we found weaker alliances with both the psychotherapist and the dietician when the ED was more severe. As expected, severe ED was associated with weaker overall alliances given ambivalence toward change and recovery and a sense of disappointment among both parties regarding treatment progress (16). Depression and anxiety (according to the DASS score) were negatively associated with patients’ alliance with the dietician but not the psychotherapist. In previous studies, higher congruence has been linked with general mental symptomatic relief (45). Our results, however, suggest that symptom-focused nutritional counseling may be less able to encompass anxiety and depression elements that interfere with the active stance, whereas psychotherapy is more suited for the containment of psychopathological symptoms, and the alliance can be maintained. Relatedly, general psychopathology and anger–hostility have recently been identified as predictors of dropout from ED intensive treatment programs, highlighting both the challenge and importance of TA maintenance enabling treatment compliance (46).

The strengths of our study lie in the novelty of exploring and comparing the alliance in therapeutic relationships with psychotherapists and dieticians and the use of validated assessment instruments. Several important limitations should be noted. Our sample size was relatively small, and the study was cross-sectional. We also did not consider confounding variables in the correlation analyses, which could be done in larger samples. In addition, data were collected during the COVID-19 pandemic amid unique pandemic-related circumstances and a massive shift to online treatment, and we did not compare data in a non-COVID-19 setting. In the study population, we grouped together patients with different ED diagnoses, an approach that is supported by the ED transdiagnostic model (47). The TA among patients and ED multidisciplinary teams should be further explored in larger longitudinal studies in order to delineate which TA relationship is the most predictive of treatment outcome. In larger studies, differences between ED diagnoses could potentially be depicted. Future studies could demonstrate whether our results are replicated in “routine” circumstances.

Our findings highlight essential clinical implications for interventions. Given the stronger congruence in the patient–dietician relationship compared to the patient–psychotherapist relationship, adopting focused psychological treatment modalities, such as CBT (48) or metacognitive therapy for ED (49, 50), wherein the therapist and the patient collaboratively establish treatment goals, and there is a mutual agreement on the objectives and direction of treatment, has the potential to improve a shared understanding between the therapist and the patient and hence improve the TA. Placing the focus on symptom reduction and dysfunctional thoughts/emotions related to body image and food may help improve all components of the TA (including goal, bond, and task), as well as the TA agreement between the patient and his/her professional helper (i.e., psychotherapists and dieticians). In CBT (48), the emphasis is on setting clear, achievable goals that both the therapist and the patient agree upon, which involves identifying specific behaviors, thoughts, or emotions to target during therapy. Similarly, metacognitive therapy (49, 50) involves recognizing and modifying dysfunctional metacognitive beliefs and processes. In this context, setting treatment goals involves agreeing upon specific metacognitive patterns to address. Moreover, pharmacological therapy (51), in combination with these psychological interventions, can help address underlying comorbid conditions such as depression or anxiety, potentially strengthening the effectiveness of the treatment strategies and improving the TA formed between the patient and his/her psychotherapist and dietician.

## Conclusion

The TA formed between patients with EDs and their psychotherapists and dieticians in the online setting during the COVID-19 pandemic was found to be relatively strong. There was a stronger match in TA observed in the patient–dietician relationship compared to the patient–psychotherapist relationship. Our findings endorse the employment of focused psychological treatment modalities for EDs, where clear goals can be agreed upon between the patient and the therapist. The differences in TA among multidisciplinary ED teams highlight the need for ongoing staff discussions to support the treatment course. Furthermore, longitudinal studies are necessary to explore the role of TA in ED multidisciplinary teams as a predictor of treatment outcomes.

## Data availability statement

The raw data supporting the conclusions of this article will be made available by the authors upon reasonable request, without undue reservation.

## Ethics statement

This study, with human participants, was approved by the Shalvata Mental Health Center’s Institutional Review Board. The study was conducted in accordance with the local legislation and institutional requirements. Written informed consent for participation in this study was provided by the participants themselves or by legal guardians/next of kin in case of minors.

## Author contributions

RE-B: Conceptualization, Methodology, Data curation, Formal analysis, Software, Writing – review & editing. RG-S: Conceptualization, Investigation, Methodology, Validation, Writing – review & editing. EZ: Conceptualization, Investigation, Methodology, Validation, Writing – review & editing. YDL: Conceptualization, Methodology, Investigation, Validation, Writing – original draft, review & editing.
